# Expression, functional role, and mechanistic insights into tRF-His-GTG-008 in lung adenocarcinoma

**DOI:** 10.1186/s12957-026-04329-z

**Published:** 2026-04-22

**Authors:** Lilin Luo, Zhengbo Long, Juanjuan Zhang, Yixing Wang, Hui Yang, Long Yang, Li Wang, Wanpu Wang

**Affiliations:** 1 Department of Pathology, The First People’s Hospital of Yunnan Province, No. 157 Jinbi Road, Xishan District, Kunming, Yunnan 650032 China; 2 The Affiliated Hospital of Kunming University of Science and Technology, Kunming, Yunnan 650032 China; 3 Kunming University of Science and Technology, Kunming, Yunnan 650031 China

**Keywords:** LATS2, Lung adenocarcinoma, tRF-His-GTG-008, tRNA derived fragments (tRFs), Tumor cell phenotype

## Abstract

**Objective:**

Transfer RNA-derived fragments (tRFs) represent a class of non-coding RNAs, typically ranging from 16 to 40 nucleotides in length, and have been implicated in the regulation of oncogenic processes. Despite emerging evidence of their involvement in various malignancies, the role of tRFs in lung adenocarcinoma remains unclear. The aim of this study is to investigate the expression levels, functional role, and molecular mechanisms of tRF-His-GTG-008 in lung adenocarcinoma.

**Methods:**

tRF-His-GTG-008, a 31-nucleotide fragment derived from the D-loop at the 5′ end of tRNA-His-GTG-1-1, was identified through high-throughput sequencing. Its expression was assessed in lung adenocarcinoma tissues and cell lines (A549, H-1975) using quantitative reverse transcription polymerase chain reaction (qRT-PCR), and its diagnostic utility was evaluated using receiver operating characteristic curve. Functional assays—including cell proliferation, clone formation, migration, and invasion experiments—were conducted following overexpression or knockdown of tRF-His-GTG-008. In vivo tumorigenicity was evaluated using a xenograft model in immunodeficient mice. The potential target gene was predicted using miRanda and TargetScan databases, and its interaction with LATS2 was validated through dual-luciferase reporter assays, RT-qPCR and western blot analysis.

**Results:**

tRF-His-GTG-008 was significantly upregulated in lung adenocarcinoma tissues and cell lines (AUC = 0.717). Overexpression of tRF-His-GTG-008 promoted cell proliferation, clone formation, migration, and invasion of A549 and H-1975 cells, whereas knockdown suppressed these oncogenic phenotypes without notable effects on the cell cycle or apoptosis. In vivo, knockdown of tRF-His-GTG-008 resulted in reduced tumor volume and weight, accompanied by decreased expression of Ki-67 and MMP9. Mechanistically, tRF-His-GTG-008 was found to directly bind to the 3’-UTR of the LATS2 gene, leading to its downregulation.

**Conclusion:**

tRF-His-GTG-008 is overexpressed in lung adenocarcinoma and demonstrates diagnostic potential. Its oncogenic effects are mediated through direct targeting and suppression of LATS2, indicating its possible utility as a novel therapeutic target in the management of lung adenocarcinoma.

**Supplementary Information:**

The online version contains supplementary material available at 10.1186/s12957-026-04329-z.

## Introduction

Lung cancer remains the leading cause of cancer-related mortality worldwide, accounting for approximately 18% of all cancer-related deaths [1]. The majority of individuals diagnosed with lung cancer present with advanced-stage disease, and recurrence or local metastasis frequently occurs despite treatment, posing significant threats to survival and overall health. Lung adenocarcinoma (LUAD) is the most prevalent histological subtype of lung cancer, comprising approximately 40% of all cases globally [2]. 

Advancements in medical technology have led to significant progress in the treatment of LUAD through radiotherapy, chemotherapy, targeted therapy, and immunotherapy. However, the prognosis remains unsatisfactory, with a five-year survival rate ranging from 15% to 25%, and as low as 5% for individuals with metastatic disease [3]. The pathogenesis of LUAD is regulated by multiple factors, underscoring the need for reliable tumor biomarkers and a deeper understanding of its molecular mechanisms to facilitate early diagnosis and therapeutic development.

The emergence of high-throughput sequencing technology has enabled the identification of numerous non-coding RNAs (ncRNAs). Small non-coding RNAs (sncRNAs), which range in length from 18 to 200 nucleotides, include miRNA, siRNA, snoRNA, snRNA, piRNA and tsRNA [4]. These molecules are widely distributed across various tissues and cell types and have garnered significant attention in cancer research, as well as in neurological and cardiovascular diseases [5–7]. Notably, sncRNAs have been demonstrated to play critical regulatory roles in tumor development and progression, further highlighting their potential as diagnostic and therapeutic targets in malignancies.

Recent studies have demonstrated that the expression of tRNA-derived fragments (tRFs), which range in length from 16 to 40 nucleotides (nt) and originate from precursor or mature tRNA, are dysregulated in various malignant tumors [8, 9]. Based on the cleavage sites of specific endonucleases, tRFs are classified into distinct subclasses: tRF-5 and tRF-3 are generated from the 5′ and 3′ ends of mature tRNAs by Dicer or angiogenin (ANG) cleavage within the D-loop and T-loop regions, respectively; tRF-1 is derived from the 3′ trailer sequence of pre-tRNA following RNase Z-mediated cleavage; tRF-2 originates from the anticodon loop under stress-induced ANG activity; and i-tRF (intermediate tRF) comprises fragments spanning multiple functional domains of tRNA. These molecules exhibit diverse and context-dependent functions in tumor cell processes—some tRFs function as tumor suppressors by blocking cell cycle progression at G1/S or G2/M checkpoints and promoting apoptosis through caspase activation or mitochondrial dysfunction, whereas others display oncogenic properties by enhancing proliferation, inhibiting apoptosis, and facilitating metastasis via epithelial-mesenchymal transition [10, 11]. 

In terms of mechanism, tRFs function as both tumor suppressor and oncogenic factors. As tumor suppressors, certain tRFs bind to Y-box binding protein 1 (YBX1) to form a transcriptional displacement model, thereby replacing oncogenic factors at the YBX1 binding site and inhibiting tumor invasion. Conversely, oncogenic tRFs have been shown to target ribosomal protein L27A (RPL27A), inducing epithelial-mesenchymal transition (EMT) and cancer stem cell (CSC) phenotypes in breast cancer cells, thereby promoting tumor progression [12, 13]. 

Structurally and functionally, tRFs exhibit similarities to miRNAs, but their gene regulatory mechanisms are more complex. In addition, tRFs are characterized by higher expression levels and greater stability compared to miRNAs, highlighting their potential as high-sensitivity tumor biomarkers [14]. Multiple mechanisms have been proposed to explain the biological functions of tRFs. Some tRFs bind to ribosomes and inhibit protein translation; [15] while others are incorporated into the Argonaute protein complex, to form a silent complex, leading to the degradation of the target mRNA [16]. Additionally, certain tRFs regulate the cell cycle process; [10] whereas others directly interact with target genes to promote tumor progression [17]. 

LATS tumor suppressor 2 (LATS2), a member of the LATS family, is an important tumor suppressor gene located on human chromosome 9q22.3 [18]. LATS2 plays a regulatory role in cell cycle-related proteins and contributes to the control of cell division. In addition, LATS2 is closely related to the Hippo signaling pathway, where it primarily regulates cell proliferation and apoptosis. Deletion or mutation of this gene has been implicated in the pathogenesis of the occurrence of breast cancer, liver cancer, and several other malignancies [19]. 

In the present study, high-throughput sequencing was utilized to identify tRF-His-GTG-008, a molecule that exhibited high expression in lung adenocarcinoma. Its expression was subsequently validated through in vivo and in vitro experiments, demonstrating its potential utility in distinguishing lung adenocarcinoma. Bioinformatics analyses were employed to screen and verify the downstream binding gene LATS2 of tRF-His-GTG-008. The findings indicated that LATS2 expression was downregulated in lung adenocarcinoma tissues and cells, and a negative regulatory relationship was observed between the carcinogenic molecule tRF-His-GTG-008 and the tumor suppressor gene LATS2. These results suggest that tRF-His-GTG-008 may competitively bind to LATS2, thereby inhibiting its expression and promoting the proliferation, clone formation, invasion, and migration of lung adenocarcinoma cells.

## Materials and methods

### Clinical samples

A total of 47 pairs of lung adenocarcinoma and corresponding adjacent non-tumor tissues (ANT) were collected. These samples were obtained from frozen tissues embedded in low-melting-point paraffin at the Department of Pathology, First People’s Hospital of Yunnan Province. Only invasive lung adenocarcinoma samples were included in the study. For each lung adenocarcinoma case, adjacent tissues were confirmed as tumor-negative and collected from a site 3–5 cm away from the tumor center. Tumor samples in which neoplastic tissue accounted for at least 80% of the total tissue composition were selected for total RNA extraction.

Following RNA extraction, the absorbance ratio at 260/280 nm was measured to determine purity and concentration. The extracted RNA was immediately subjected to reverse transcription and quantitative real-time polymerase chain reaction (RT-qPCR) analysis. The remaining RNA samples were stored at -80 °C for future use.

All patients enrolled in this study had not received any form of treatment prior to surgery. The collection and use of lung adenocarcinoma and paraneoplastic paraffin tissues were approved by the Ethics Committee of the First People’s Hospital of Yunnan Province (Approval number: KHHL2022-KY037). All patients and their families were provided with detailed information regarding the study, and written informed consent was obtained before participation.

### Cell culture

The Beas-2B normal human lung epithelial cell line, lung adenocarcinoma cell lines (A549, H-1975 and PC9), and the H-1299 non-small cell lung cancer cell were obtained from Wuhan Pricella Biotechnology Co., Ltd. (China). Cells were cultured in RPMI-1640 or DMEM (Gibco) supplemented with 10% fetal bovine serum and 1% penicillin/streptomycin. Incubation was carried out at 37℃ in a humidified atmosphere containing 5% CO_2_. Cells were passaged once the growth density reached 80%–90%, and all the cells used in the experiment were within 10 generations after resuscitation.

### Cell transfections

The A549 and H-1975 cells were seeded and transfected once cell density reached 70%–80% confluence. The synthesized tiRNA single-chain mimetic/inhibitor and the corresponding negative control were introduced using Lipofectamine 3000 transfection reagent (Invitrogen), following the manufacturer’s instructions. The tRF-His-GTG-008 mimic and negative control (Ribobio, China) were optimized for transfection in A549 and H-1975 cells. Pre-experimental optimization determined that the optimal transfection concentration for the mimic was 50 nM, while the optimal concentration of the inhibitor was 100 nM. Subsequent transfections were performed at these concentrations.

### CCK8 assay

Cell viability was assessed using the Cell Counting Kit-8 (CCK-8) from MedChemExpress (USA). Cells were inoculated into 96-well plates at a density of 5 × 10^3^ and cultured at 37℃ with 5%CO_2_ for 4 to 6 h until the cells adhere to the plate completely for transfection. After transfection, the absorbance at 450 nm was measured at 0, 24, 48 and 72 h. All CCK-8 assays were conducted under dark conditions. A CCK-8 working solution, comprising 10% of the complete culture medium, was added to each well, and incubation was carried out in the dark for 2–4 h at 37℃ with 5% CO_2_ before absorbance detection using a microplate reader.

### Colony formation assay

Cells were inoculated into 6-well plates at the density of 0.5 × 10^3^ to 1 × 10^3^ cells per well and incubated for six days until visible cell colonies formed. The cells were then fixed with 4% paraformaldehyde and stained with 1% crystal violet. Colony numbers were quantified using an optical microscope.

### Transwell assay

#### Invasion assay

Following transfection, cells were digested, resuspended in serum-free culture and inoculated into the upper chamber of a Transwell system (Coring Inc costar^®^, USA). A total of 400 µL of medium containing 10% FBS was added to the lower chamber. After incubation at 37℃ for 24 h, non-migrated cells on the upper side of the filter membrane were removed using a cotton swab. Cells that had invaded the lower surface were subsequently fixed, stained and quantified.

#### Migration assay

The transfected cells were digested, resuspended in serum-free culture medium, and inoculated into the upper chamber of Transwell system (Coring^®^ Matrigel^®^ invasion chamber, USA) without Matrigel coating. The subsequent procedural steps were identical to those of the invasion assay.

### Real-time qPCR analysis

Primers used for RT-qPCR (Table) were synthesized by Tsingke (China). The miRNA Universal SYBR qPCR Master Mix was purchased from Vazyme (China). Total RNA was extracted from the cells to synthesize complementary DNA strands. RT-qPCR was performed using a premixed reaction system, and relative gene expression levels were quantified using the 2-∆∆Ct method.

### Dual‑luciferase reporter assay

The full-length 3′-UTR sequences of POGK, ARHGEF39, and LATS2, containing either wild-type or mutant putative tRF-His-GTG-008 binding sites, were cloned downstream of the firefly luciferase gene in the pGL3-Control vector (Promega), which contains an SV40 promoter upstream of the reporter gene. The pRL-SV40 vector expressing Renilla luciferase was co-transfected as an internal control for normalization. The 293T cells were co-transfected with Lipofectamine TM 2000 (Invitrogen, USA) and either a tRF-His-GTG-008 overexpression vector or mimic vector. Following 48 h of incubation, firefly and sea kidney luciferase activities were measured using a fluorescent microplate reader (Agilent, USA), according to the manufacturer’s protocol.

### Western blot analysis

Primary antibodies, including Goat Anti-Mouse IgG-HRP and Goat Anti Rabbit IgG-HRP, were purchased from Abmart (USA), while the secondary antibody, mouse anti-β-actin monoclonal antibody, was purchased from ZSGB-BIO (China). Total cellular protein was extracted, and its concentration was determined using a Bicinchoninic Acid (BCA) protein assay kit. Protein samples were denatured at 100 °C for 10 min. A total of 20 µg of protein was loaded onto a Sodium Dodecyl Sulfate-Polyacrylamide Gel Electrophoresis (SDS-PAGE) gel along with a pre-dyed protein marker (Thermo, #26616). Following electrophoretic separation, proteins were transferred onto a polyvinylidene difluoride (PVDF) membrane (Immobilon, China). The membrane was then subjected to blocking, overnight incubation with primary antibodies, washing, incubation with secondary antibodies, and additional washing steps. Protein bands were visualized using a chemiluminescence detection method. The intensity of the bands was quantified using NIH ImageJ software (version 1.43).

### Animal experiment

The animal experiment protocol was approved by the Kunming Medical University Animal Ethics Review Committee (Approval number: kmmu20241884). Female BALB/c nude mice (6 weeks old, weighing 18–20 g) were obtained from the university’s Experimental Animal Center (Animal License No.: SCXK (Dian) 2020-0004). Animals were housed under SPF-grade barrier conditions with ad libitum access to sterile water and SPF-grade irradiated feed. For euthanasia, mice were anesthetized via inhalation of 4% isoflurane followed by cervical dislocation. The nude mouse experiment included three groups (*n* = 3 per group): Control; antagomir-NC (negative control with scrambled sequence); and antagomir. A total of nine nude mice were used, randomly selected from multiple cages for assignment to these groups.

The tRF-antagomir and its corresponding negative control (NC) were synthesized by Guangzhou RiboBio Technology Co., Ltd.

### Immunohistochemistry

Ki-67 and MMP9 antibodies were purchased from Abcam (UK). Tumor tissues from nude mice were dehydrated, embedded, sliced, and subsequently dewaxed at room temperature following the procedures outlined in the immunohistochemistry protocol. The slides were scanned using digital pathology reading software, and images were analyzed with ImageJ software. Immunohistochemical results were assessed based on the proportion of positively stained cells, with three images analyzed per group. The scoring system was defined as follows: 0 points (no positive cells), 1 point (0% < number of positive cells < 25%), 2 points (25% ≤ positive cells < 50%), 3 points (50% ≤ positive cells < 75%).

### Statistical analysis

GraphPad Prism 8.0.2 software was used for graphical representation, while SPSS software was used for statistical analysis. Differences between two groups were assessed using a two-tailed paired t-test. The correlation between tRF-His-GTG-008 expression and pathological characteristics of individuals with lung adenocarcinoma was evaluated using a nonparametric test, while the Kruskal-Wallis H test was applied for comparisons among multiple groups. The receiver operating characteristic (ROC) curve was used evaluate the diagnostic potential of tRF-His-GTG-008 in lung adenocarcinoma. All statistical tests were two-tailed, and *p* < 0.05 was considered statistically significant. Effect sizes (Cohen’s d for continuous variables) and 95% confidence intervals (95% CI) were calculated using bootstrap resampling (1000 iterations). Mean differences with 95% CI are reported for inter-group comparisons.

## Results

### High expression of tRF-His-GTG-008 in lung adenocarcinoma cells and tissues

**Fig. 1:**
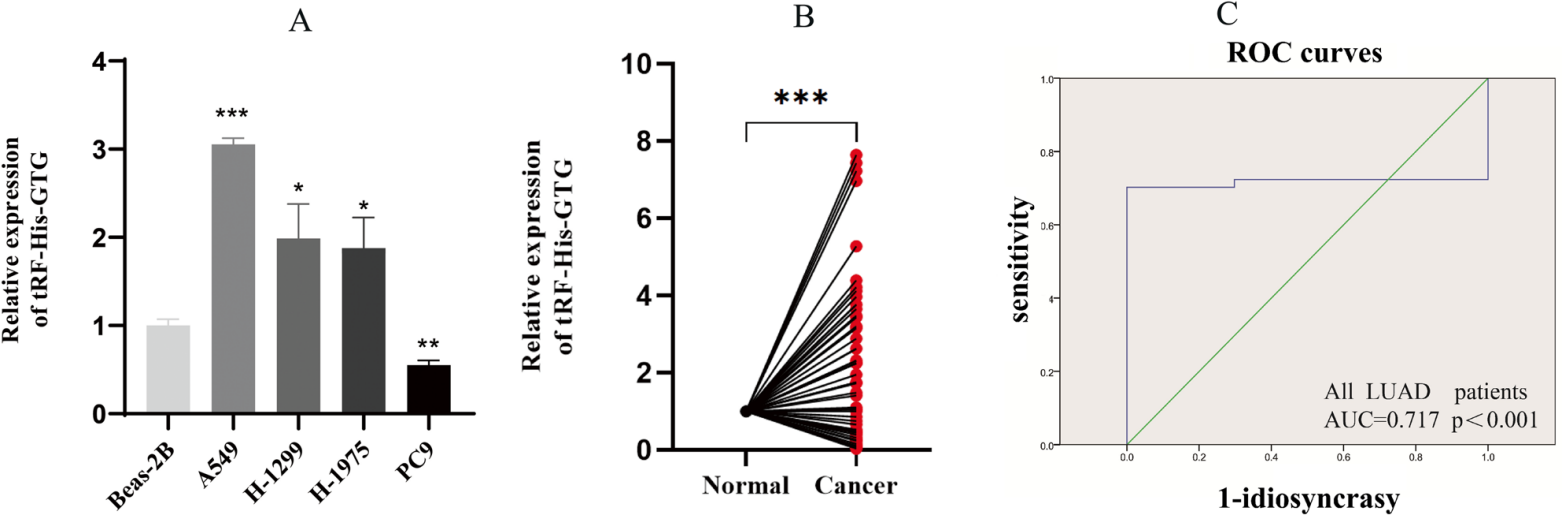
Expression profile of tRF-His-GTG-008. **a** Expression level of tRF-His-GTG-008 in lung adenocarcinoma cells. **b** Expression level of tRF-His-GTG-008 in lung adenocarcinoma tissues versus adjacent non-cancerous tissues. **c** Receiver Operating Characteristic (ROC) curve analysis of tRF-His-GTG-008 (ns: not significant, *P < 0.05, **P < 0.01, ***P < 0.001, *n* = 3)

High-throughput tRF & tiRNA chip sequencing revealed tRF-His-GTG-008 to be highly expressed in lung adenocarcinoma. This fragment, 31 nucleotides in length, originates from the D-loop at the 5’ end of mature tRNA-His-GTG-1-1 and has the sequence: 5’-GCCGUGAUCGUAUAGUGGUUAGUACUCUGCG-3’. RT-qPCR analysis demonstrated that the expression level of tRF-His-GTG-008 was significantly elevated in non-small cell lung cancer (NSCLC) cell lines (H-1299, A549, PC9, H-1975) compared to the human normal lung epithelial cell line Beas-2B (Fig. 1a). Furthermore, the expression level of tRF-His-GTG-008 in lung adenocarcinoma tissues (*n* = 47) was significantly higher than in the corresponding adjacent non-tumor tissues (*n* = 47) (Fig. 1b). ROC curve analysis showed that the area under the curve (AUC) was 0.717, *p* < 0.001 (Fig. 1c), suggesting that tRF-His-GTG-008 may serve as a potential diagnostic biomarker for lung adenocarcinoma.

### tRF-His-GTG-008 promotes proliferation of lung adenocarcinoma cells

**Fig. 2:**
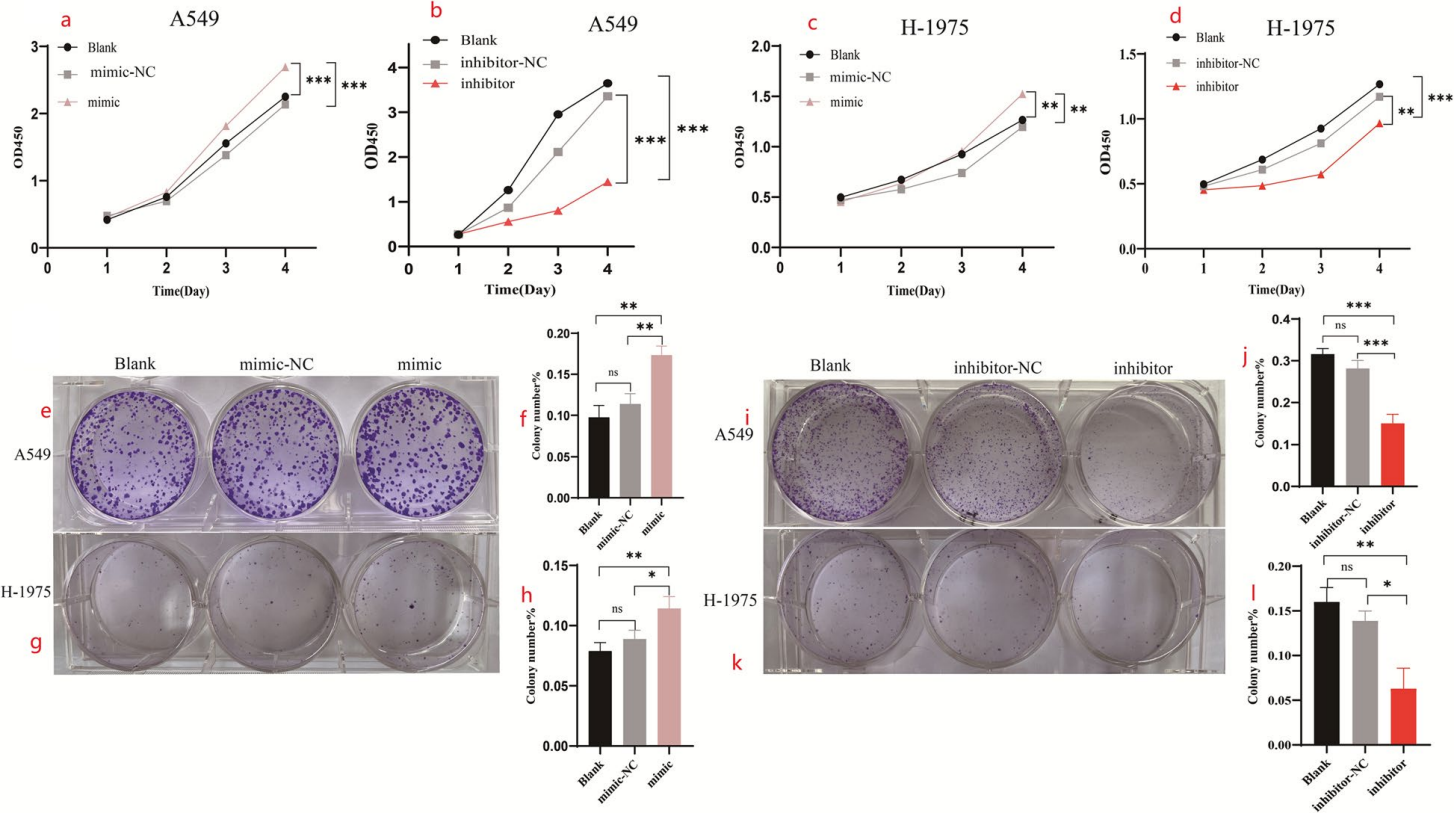
Effects of tRF-His-GTG-008 overexpression and knockdown on proliferation and colony formation in lung adenocarcinoma cells. **a**-**d** Overexpression of tRF-His-GTG-008 promotes proliferation of lung adenocarcinoma cells, while knockdown of tRF-His-GTG-008 suppresses proliferation. **e**-**l** Overexpression of tRF-His-GTG-008 enhances colony formation in lung adenocarcinoma cells, whereas knockdown of tRF-His-GTG-008 inhibits colony formation (ns: not significant, *P < 0.05, **P < 0.01, ***P < 0.001, *n* = 3)

To evaluate the biological function of tRF-His-GTG-008 in lung adenocarcinoma, gain- and loss-of-function experiments were conducted in A549 and H-1975 cells. CCK-8 analysis demonstrated that the overexpression of tRF-His-GTG-008 significantly promoted cell proliferation, whereas its knockdown led to a marked reduction in proliferation (Fig. 2a-d).

### tRF-His-GTG-008 promotes clone formation in lung adenocarcinoma cells

The effect of tRF-His-GTG-008 on clonogenic potential was assessed by overexpressing and inhibiting tRF-His-GTG-008 in lung adenocarcinoma cells, followed by evaluation of colony formation over six days (Fig. 2e-l). The results indicated that tRF-His-GTG-008 overexpression significantly enhanced colony formation, whereas its inhibition led to a marked reduction in clonogenic capacity.

### tRF-His-GTG-008 promotes migration and invasion of lung adenocarcinoma cells

**Fig. 3:**
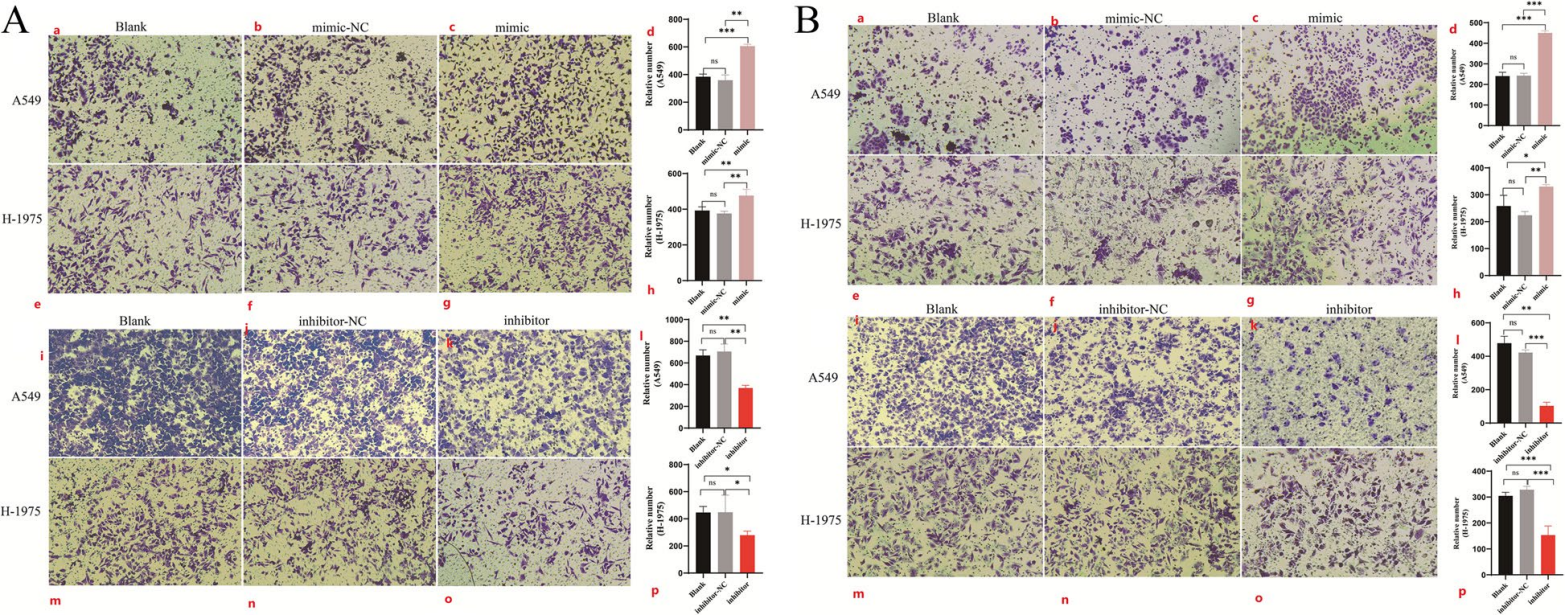
Effects of tRF-His-GTG-008 Overexpression and Knockdown on Migration and Invasion Capacities of Lung Adenocarcinoma Cells. (Fig. 3**A** a-h) Overexpression of tRF-His-GTG-008 promotes migration of lung adenocarcinoma cells. (Fig. 3**A** i-p) Knockdown of tRF-His-GTG-008 suppresses migration of lung adenocarcinoma cells. (Fig. 3**B** a-h) Overexpression of tRF-His-GTG-008 enhances invasion of lung adenocarcinoma cells. (Fig. 3**B** i-p) Knockdown of tRF-His-GTG-008 inhibits invasion of lung adenocarcinoma cells

To investigate the role of tRF-His-GTG-008 in cell migration and invasion, a Transwell assay was performed to assess the migratory and invasive potential of lung adenocarcinoma cells. The number of migrating and invading cells was significantly higher in the tRF-His-GTG-008 overexpression group compared to the control group. Conversely, treatment with a tRF-His-GTG-008 inhibitor effectively reversed these effects (Fig. 3A and B).

### Knockdown of tRF-His-GTG-008 inhibits tumor growth in nude mice

**Fig. 4:**
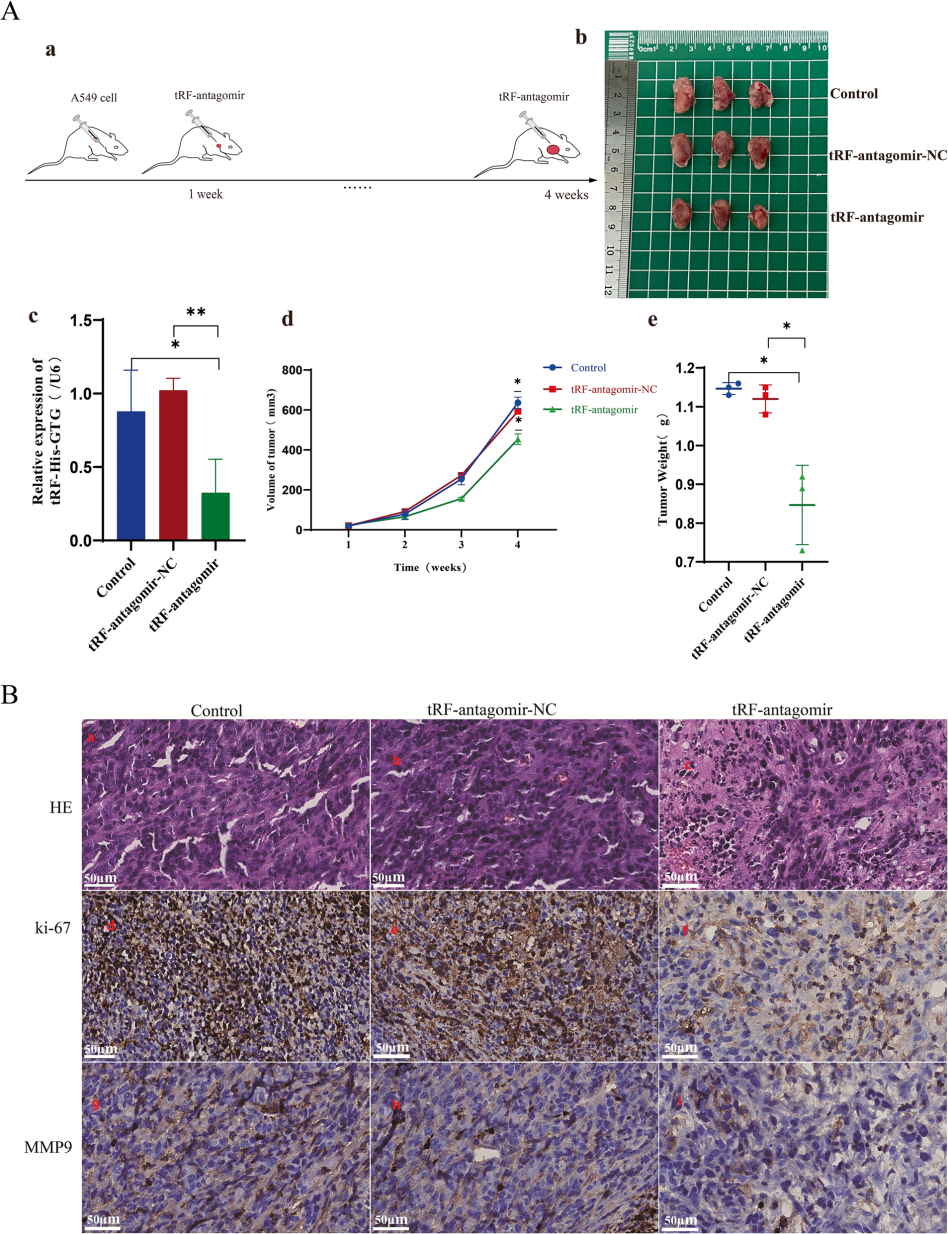
Effects of tRF-His-GTG-008 Knockdown on Tumor Growth in Nude Mice. (Fig. 4**A** a) Establishment of a xenograft tumor model in nude mice. (Fig. 4**A** b) Representative images of tumor masses. (Fig. 4**A** c) Expression level of tRF-His-GTG-008 in tumor tissues. (Fig. 4**A** d) Tumor growth curve. (Fig. 4**A** e) Tumor weight measurement. (Fig. 4**B**) Histopathological (H&E) and immunohistochemical (IHC) staining of tumor tissues (*P < 0.05, **P < 0.01, *n* = 3)

To investigate the role of tRF-His-GTG-008 in vivo, A549 cells were cultured, prepared as a cell suspension, and injected into the subcutaneous of nude mice (three mice per group), and tumor volume was measured using a digital caliper every 3 days with the formula V = 0.5 × L × W² (where L = longest diameter, W = shortest diameter). Once tumor formation was observed, tRF-antagomir was administered every two days. Body weight and tumor size were measured throughout the experiment. After four weeks, tumors were excised (Fig. 4Aa, Fig. 4Ab), and the expression level of tRF-His-GTG-008 was assessed using RT-qPCR (Fig. 4Ac). Tumor volume was significantly reduced in the tRF-antagomir group (0.32 ± 0.05 cm³) compared to the control (0.65 ± 0.10 cm³; mean difference = -0.33 cm³, 95% CI: -0.45 to -0.21, *p* < 0.001) and tRF-antagomir-NC groups (0.62 ± 0.08 cm³; mean difference = -0.30 cm³, 95% CI: -0.42 to -0.18, *p* < 0.001) (Fig. 4Ad). Similarly, tumor weight was markedly lower in the tRF-antagomir group (0.19 ± 0.03 g) versus control (0.38 ± 0.06 g; mean difference = -0.19 g, 95% CI: -0.27 to -0.11) and NC groups (0.36 ± 0.05 g; mean difference = -0.17 g, 95% CI: -0.24 to -0.10) (Fig. 4Ae).

Histological analysis was performed using hematoxylin and eosin (HE) staining, and immunohistochemical detection of Ki-67 (a tumor proliferation marker) and MMP9 (an invasion-associated marker) was conducted. Ki-67 positive cell rate was 45.6 ± 6.8% in the tRF-antagomir group, significantly lower than in control (82.4 ± 5.2%; mean difference = -36.8%, 95% CI: -47.1 to -26.5, Cohen’s d = 6.2) and NC groups (80.1 ± 4.7%; mean difference = -34.5%, 95% CI: -44.3 to -24.7). MMP9 expression scores (0–3 scale) were reduced in the tRF-antagomir group (1.00 ± 0.00) compared to control (2.67 ± 0.58; mean difference = -1.67, 95% CI: -2.25 to -1.09) and NC groups (2.33 ± 0.58; mean difference = -1.33, 95% CI: -1.88 to -0.78) (Fig. 4B; Table 1).

**Table 1:** Scale scores of Ki-67 and MMP 9 positive cells

Score	Control			tRF-antagomir-NC			tRF-antagomir		
	P1	P2	P3	P1	P2	P3	P1	P2	P3
Ki-67	3	3	3	3	3	2	1	1	1
MMP9	2	2	2	2	2	2	1	1	1

### Binding of tRF-His-GTG-008 to the 3’ UTR of LATS2 and its role in the progression of lung adenocarcinoma

**Fig. 5:**
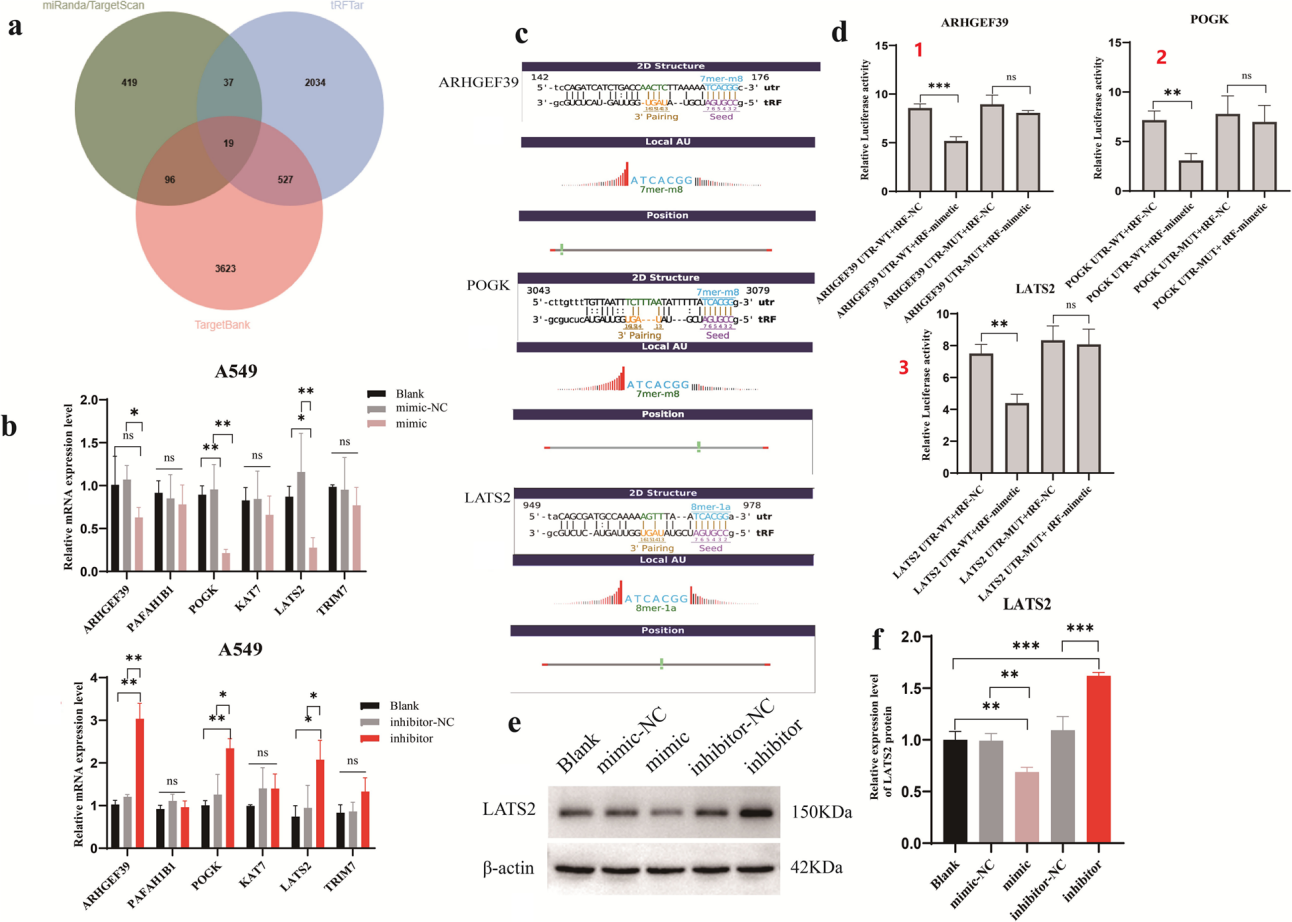
Screening and validation of downstream binding genes of tRF-His-GTG-008. **a** Potential target genes of tRF-His-GTG-008 were screened using miRanda, TargetScan, tRFTar, and TargetBank databases. **b** Expression levels of downstream genes in A549 cells following overexpression or inhibition of tRF-His-GTG-008. **c**, **d** Interaction of tRF-His-GTG-008 with the 3’UTR of *ARHGEF39*, *POGK*, and *LATS2*. **e**, **f** Western blot analysis of LATS2 protein expression levels. (ns: not significant, *P < 0.05, **P < 0.01, ***P < 0.001, *n* = 3)

To elucidate the mechanism underlying the role of tRF-His-GTG-008 in lung adenocarcinoma, a bioinformatics analysis was conducted using three mRNA target prediction databases—miRanda/TargetScan, tRFTar and TargetBank. A total of 19 overlapping genes were identified (Fig. 5a). Based on free energy score, structural score, 2D structural binding site of the seed region, and a review of relevant literature, six genes (ARHGEF39, PAFAH1B1, POGK, KAT7, LATS2, and TRIM7) were selected for further investigation.

To validate these findings, tRF-His-GTG-008 was overexpressed and knocked down in A549 cells, and the expression levels of the selected genes were assessed. Among the six candidate targets, ARHGEF39, POGK, and LATS2 exhibited significant expression changes following tRF-His-GTG-008 modulation. Notably, LATS2 mRNA and protein levels were markedly downregulated upon tRF-His-GTG-008 overexpression, whereas inhibition of tRF-His-GTG-008 led to increased LATS2 expression (Fig. 5b, e and f), consistent with the oncogenic role of tRF-His-GTG-008 in lung adenocarcinoma. (Fig. 5b).

For further validation, the 3’UTR regions of ARHGEF39, POGK, and LATS2 were cloned into the dual-luciferase vector, and a dual-luciferase reporter assay was performed. The results confirmed that tRF-His-GTG-008 directly bound to the 3’UTR of ARHGEF39, POGK and LATS2 (Figs. 5c and d). Additionally, western blot analysis revealed that LATS2 protein expression was significantly increased following tRF-His-GTG-008 overexpression, whereas inhibition of tRF-His-GTG-008 led to a reduction in LATS2 expression (Figs. 5e, f). These findings suggest that tRF-His-GTG-008 may promote lung adenocarcinoma progression by binding to the 3’ UTR of LATS2 and regulating its expression.

## Discussion

Lung adenocarcinoma is among the most lethal solid tumors, highlighting the importance of early screening and timely intervention [20]. With advancements in detection technologies, numerous lung cancer biomarkers and diagnostic molecules have been investigated, including EGFR, ALK, serum tumor markers CEA, and NSE [17, 21–23]. Additionally, miRNAs, which belong to the non-coding RNA family, have been explored as potential diagnostic biomarkers [24]. 

The tRF molecules are superior to miRNA in both expression abundance and stability, suggesting their potential as highly sensitive tumor diagnostic markers. Previous studies have demonstrated that the expression of tRF-Leu-CAG is significantly upregulated in stage IV non-small cell lung cancer, with a strong ability to distinguish this advanced stage [25]. Similarly, plasma exosomal tRF-ALa-AGC-036 has been found to be significantly associated with tumor staging and metastasis, effectively differentiating between early-stage (0 + II) and late-stage (III + IV) disease [26]. Furthermore, tRF-31-79MP9P9NH57SD is highly expressed in individuals with non-small cell lung cancer, correlating with clinical stage and lymph node metastasis, and has been proposed as potential diagnostic marker [27]. 

Researchers You et al. found that tRF-1:29-ProAGG-1-M6 and tRF-55:76-Tyr-GTA-1-M2 can be used as highly sensitive diagnostic markers for lung adenocarcinoma, and they are also significantly related to clinicopathological features such as tumor metastasis stage, lymph node stage, and carcinoembryonic antigen expression level [28]. Additionally, tRF-Gly-CCC-046, tRF-TyrGTA-010, and tRF-Pro-TGG-001 have been investigated as early diagnostic markers for breast cancer, with findings indicating significant downregulation in the serum of 214 patients with breast cancer compared to 113 healthy controls [29]. Moreover, a study by Li et al. revealed that tRF-Pro-CGG exhibited a high diagnostic accuracy for pancreatic ductal adenocarcinoma, with an AUC value of 0.92 (95% CI:0.8466–0.9948, *p* < 0.0001), a sensitivity of 75.7%, and a specificity of 93.3%. Its expression was significantly downregulated and correlated with clinical staging (*p* = 0.000) and N stage (*p* = 0.000) [30]. 

While tRF-Leu-CAG and tRF-Ala-AGC-036 have been implicated in non-small cell lung cancer progression, our study specifically identifies tRF-His-GTG-008 as a novel diagnostic biomarker with an AUC of 0.717 for LUAD, suggesting distinct clinical utility. Preliminary analysis of public databases indicates that tRF-His-GTG-008 exhibits relatively specific upregulation in LUAD compared to other malignancies, though its expression pattern across diverse cancer types warrants systematic investigation in future multi-cohort studies to definitively establish its tissue specificity.

In the present study, tRF-His-GTG-008 was identified as highly expressed in lung adenocarcinoma and adjacent tissues through high-throughput sequencing. Cellular experiments demonstrated that tRF-His-GTG-008 exhibited high expression in A549, H-1975 and H-1299 cells, whereas its expression was low in PC9 cells. In addition, the ROC curve analysis yielded an AUC value of 0.717, indicating good diagnostic performance. However, no significant correlation was observed between tRF-His-GTG-008 expression and the clinicopathological features of individuals with lung adenocarcinoma.

The tRF molecules have been implicated in the regulation of various diseases, including tumor progression, Alzheimer’s disease, and lipid metabolism, exhibiting complex regulatory functions [31–33]. A previous study reported that tRF-23-Q99P9NDD promoted proliferation, migration, and invasion of gastric cancer cells in vitro [17]. In epithelial ovarian cancer, Panoutsopoulou et al. found that 3’U-tRFs promoted the growth and metastasis of tumor cells through CCK-8 and wound healing experiments [21]. Investigations into thyroid cancer have indicated that tRF-30 inhibited the proliferation and invasion of cancer cells. Mechanistically, tRF-30 directly binds to the biotin-dependent enzyme pyruvate carboxylase (PC), interfering with the intermediate anaerobic enzyme in the tricarboxylic acid cycle, and further affecting metabolic reprogramming and cancer progress [22]. Similarly, in pancreatic cancer, overexpression of tRF-18-8R6546D2 has been found to enhance cancer cell proliferation, migration, and invasion, while also inhibiting cell apoptosis [34]. 

In the present study, in vitro functional experiments demonstrated that tRF-His-GTG-008 overexpression significantly promoted cell proliferation, colony formation, invasion, and migration, whereas inhibition of tRF-His-GTG-008 expression led to a marked reduction in these processes. Furthermore, in a tumor-bearing model using nude mice, suppression of tRF-His-GTG-008 expression resulted in reduced tumor growth.

LATS2 serves as a critical tumor suppressor gene and has been reported to interact with upstream molecules, such as miRNA to regulate oncogenic processes. The carcinogenic molecule miR-25 binds to the downstream target gene LATS2 to regulate tumor growth and metastasis [24]. Similarly, in pancreatic cancer, miR-31-5p targets LATS2, influencing the Hippo signaling pathway in pancreatic stellate cells through extracellular vesicle transfer, ultimately contributing to chemoresistance in cancer cells [35]. 

In human lung cancer cell lines, an inverse correlation has been observed between the expression levels of LATS2 and tristetraprolin (TTP), suggesting that TTP may regulate the growth of cancer cells by modulating the stability of LATS2 mRNA [36]. Moreover, LATS2 expression has demonstrated a significant association with clinicopathological features in NSCLC. Immunohistochemical analysis of 73 clinical samples of cancer tissues and 22 normal lung tissues revealed that the low-level LATS2 protein expression correlated negatively with tumor classification (T-type, *p* = 0.001), lymph node involvement (N-type, *p* = 0.005) and clinical stage (*p* = 0.001). Additionally, individuals with low LATS2 expression exhibited significantly shorter overall survival compared to those with high LATS2 expression [37]. 

The therapeutic targeting of tRFs presents significant challenges that warrant careful consideration. Delivery specificity remains a major hurdle, as antagomirs or other tRF inhibitors must efficiently reach tumor tissues while minimizing accumulation in normal organs, necessitating advanced delivery systems such as tumor-targeting nanoparticles or chemical modifications to enhance tissue selectivity. Additionally, potential off-target effects pose concerns, given the sequence similarity among tRF family members and other small non-coding RNAs, which may lead to unintended suppression of physiologically important transcripts; thus, rigorous specificity validation and safety profiling will be essential prerequisites for clinical translation of tRF-based therapeutics.

Further studies have indicated that the expression of LATS2 and YAP, key components of the Hippo pathway, may interact with the cancer suppressor gene FOXP3 in lung squamous cell carcinoma to inhibit the growth of cancer cells [38]. In renal cell carcinoma, miR-139-5p has been found to target and regulate glycosyltransferase 2 protein (GALNT2) to promote the proliferation of cancer cells, and this effect is reversed when LATS2 is activated [39]. Additionally, LATS2 has been implicated in tumor drug resistance. In non-small cell lung cancer, meta-analysis demonstrated that positive expression of Y-box binding protein 1 (YBX1) is significantly associated with poor patient prognosis. In lung adenocarcinoma, the m6A methyltransferase METTL3 has been reported to induce epigenetic inheritance of small nucleolar host gene 17 (SNHG17), leading to LATS2 suppression and promoting resistance to gefitinib [40, 41]. 

In the present study, LATS2 was identified through bioinformatics-based prediction, RT-qPCR, western blot, and dual-luciferase reporter assays. Further validation of LATS2 expression in Beas-2B and A549 cells, as well as GEPIA database analysis, demonstrated that LATS2 expression was reduced in lung adenocarcinoma tissues and cell lines. A negative regulatory relationship was observed between the oncogenic molecule tRF-His-GTG-008 and the tumor suppressor gene LATS2. These findings suggest that tRF-His-GTG-008, which is highly expressed in lung adenocarcinoma tissues and cells, may competitively bind to LATS2, thereby inhibiting its expression and promoting cell proliferation, clonal formation, invasion, and migration in lung adenocarcinoma cells.

## Conclusions

The expression level of tRF-His-GTG-008 was significantly upregulated in lung adenocarcinoma, demonstrating high diagnostic efficiency for this malignancy. The tRF-His-GTG-008 functioned as an oncogenic factor in lung adenocarcinoma and exerted its effects by binding to the 3’-UTR of the LATS2 gene, thereby influencing tumor progression.

## Supplementary Information

Supplementary Material 1.

## Data Availability

The data that support the findings of this study are available from the corresponding author, Wanpu Wang , upon reasonable request.

## Abbreviations

LUAD: Lung adenocarcinoma
tRFs: tRNA derived fragments
RPL27A: Ribosomal Protein L27A
sncRNA: Small non-coding RNAs
YBX1: Y-box binding protein 1
EMT: Epithelial-Mesenchymal Transition
CSC: Cancer Stem Cell
LATS2: LATS tumor suppressor2
RPL27A: Ribosomal Protein L27A
SNHG17: Small nucleolar host gene 17
IHC: Immunohistochemistry
HE: hematoxylin-eosin staining
