# Supplementary figures and images for: Expression, functional role, and mechanistic insights into tRF-His-GTG-008 in lung adenocarcinoma
Source: World J Surg Oncol. 2026 Apr 22;24:242. doi: 10.1186/s12957-026-04329-z (PMC13237919; doi:10.1186/s12957-026-04329-z)

LATS2

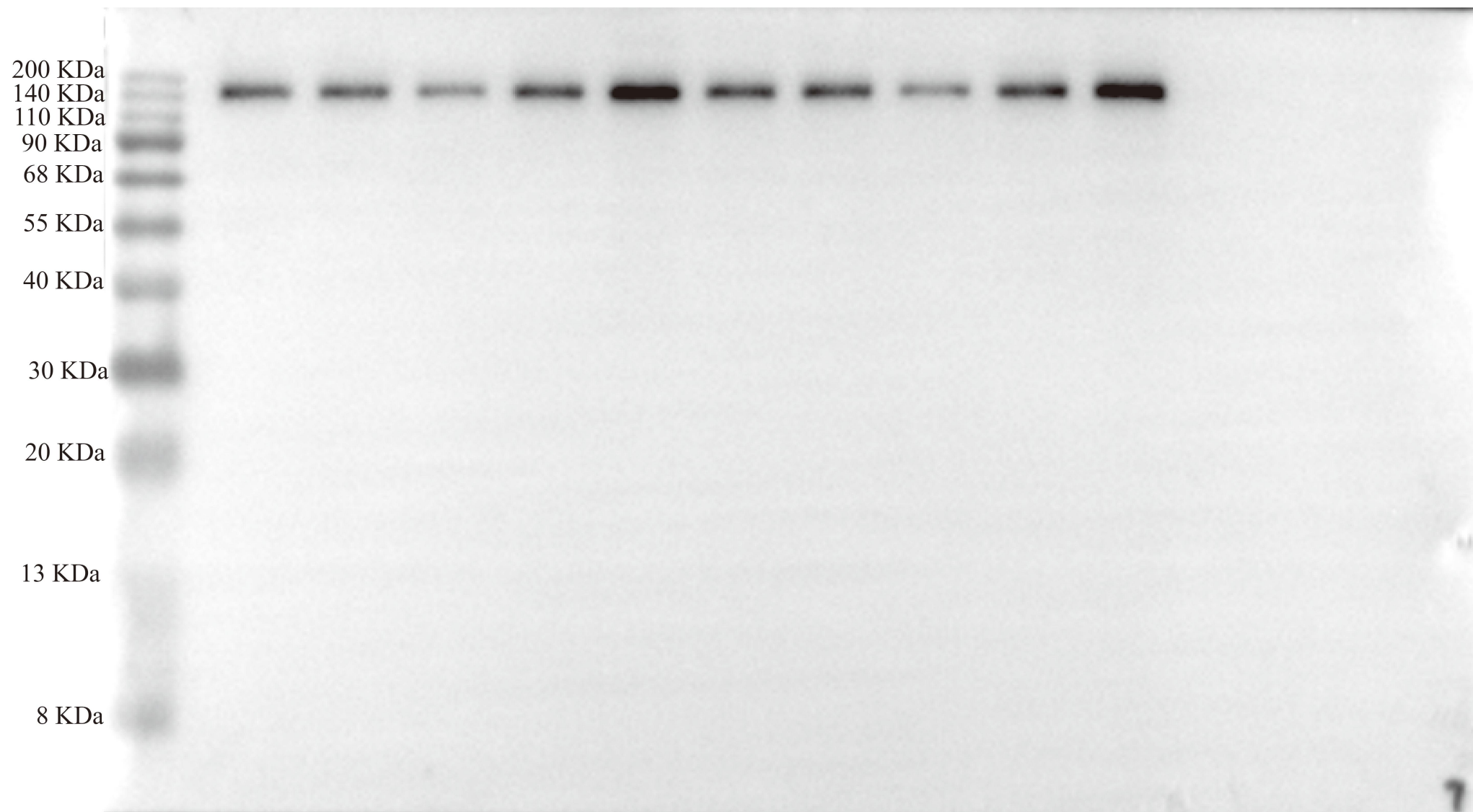

-actin

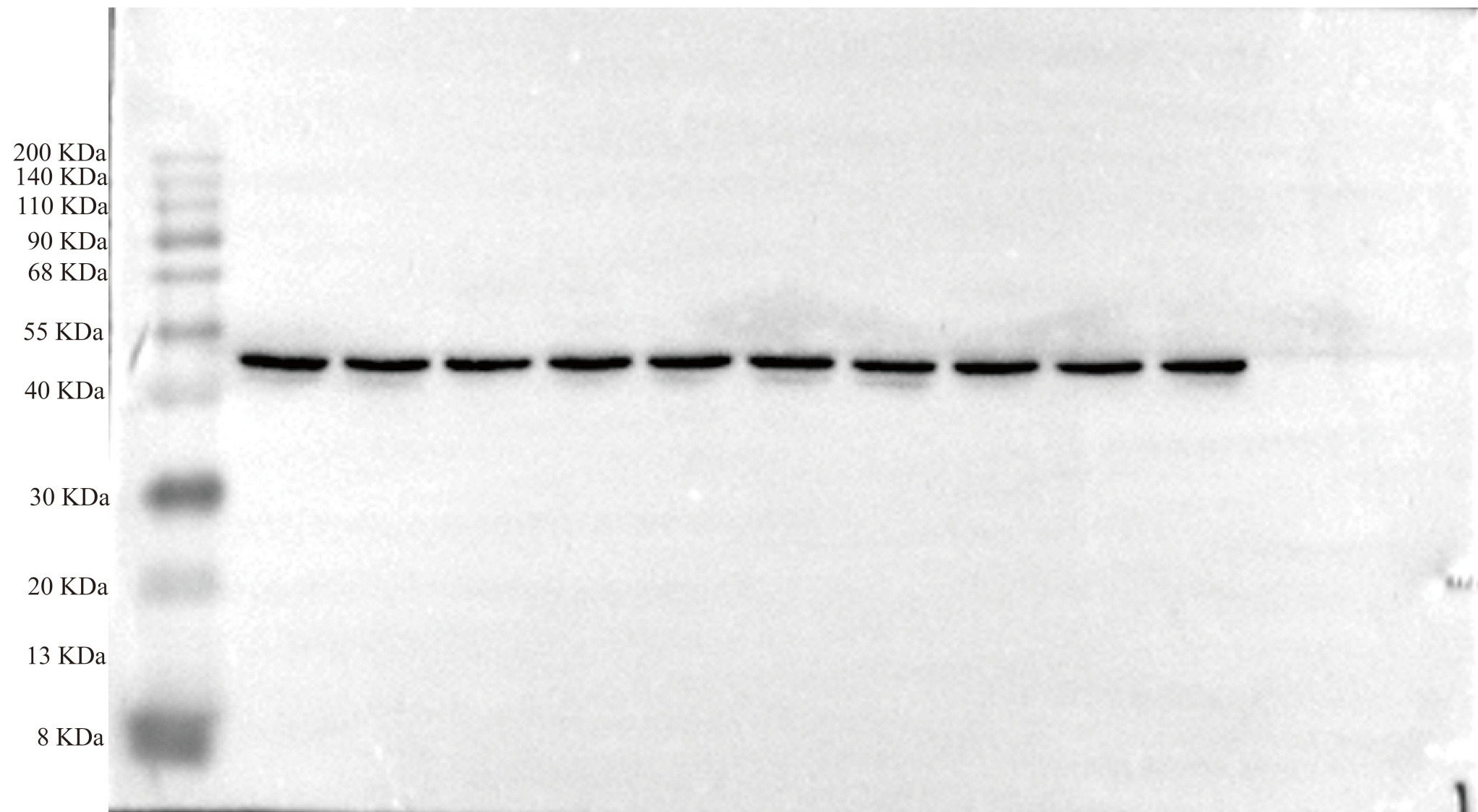

Supplement: Supplementary file 1 — Supplementary Material 1. [file 12957_2026_4329_MOESM1_ESM.pdf]
